# Machine Learning Magnetic Parameters from Spin Configurations

**DOI:** 10.1002/advs.202000566

**Published:** 2020-07-01

**Authors:** Dingchen Wang, Songrui Wei, Anran Yuan, Fanghua Tian, Kaiyan Cao, Qizhong Zhao, Yin Zhang, Chao Zhou, Xiaoping Song, Dezhen Xue, Sen Yang

**Affiliations:** ^1^ MOE Key Laboratory for Nonequilibrium Synthesis and Modulation of Condensed Matter School of Science State Key Laboratory for Mechanical Behavior of Materials Xi'an Jiaotong University Xi'an 710049 China; ^2^ Key Laboratory of Optoelectronic Devices and Systems of Ministry of Education and Guangdong Province College of Optoelectronic Engineering Shenzhen University Shenzhen 518060 China; ^3^ Key Laboratory of Intelligent Perception and Image Understanding of Ministry of Education International Research Center for Intelligent Perception and Computation Joint International Research Laboratory of Intelligent Perception and Computation School of Artificial Intelligence Xidian University Xi'an 710071 China

**Keywords:** machine learning, micro‐magnetism, parameter estimation, spin configurations

## Abstract

Hamiltonian parameters estimation is crucial in condensed matter physics, but is time‐ and cost‐consuming. High‐resolution images provide detailed information of underlying physics, but extracting Hamiltonian parameters from them is difficult due to the huge Hilbert space. Here, a protocol for Hamiltonian parameters estimation from images based on a machine learning (ML) architecture is provided. It consists in learning a mapping between spin configurations and Hamiltonian parameters from a small amount of simulated images, applying the trained ML model to a single unexplored experimental image to estimate its key parameters, and predicting the corresponding materials properties by a physical model. The efficiency of the approach is demonstrated by reproducing the same spin configuration as the experimental one and predicting the coercive field, the saturation field, and even the volume of the experiment specimen accurately. The proposed approach paves a way to achieve a stable and efficient parameters estimation.

## Graphical abstract

Magnetic parameters can be efficiently estimated from an experimental observation of spin configuration by combining machine learning and micro‐magnetic simulation. The method includes learning a mapping from spin configurations to magnetic parameters on a small amount of micro‐magnetic simulated images and applying the trained machine learning model to a single unexplored experimental image to estimate its key parameters.

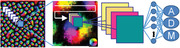

## 1 Introduction

Theoretical models describe the underlying physics of a given physical system and are able to understand and predict properties of a particular system if the model parameters are estimated appropriately.^[^ ^1^ ^]^ A typical example is the micro‐magnetic model which evolves the spin configurations to the stable state according to the magnetic Hamiltonian.^[^ ^2^ ^]^ The magnetic Hamiltonian is an operator corresponding to the total energy of the magnetic system, which usually includes several terms. Specifically, the Heisenberg exchange energy tries to align neighboring spins; the Dzyaloshinskii–Moriya interaction favors the canting of neighboring spins; the Zeeman energy aligns the spin with the external magnetic field; the magnetostatic energy tries to close all the flux loop to decrease stray fields outside of the magnet; the magnetocrystalline anisotropy energy intends to align the spin with the anisotropy direction or perpendicular to the anisotropy axis. The strength of these contributions is determined by parameters such as the Heisenberg exchange stiffness (*A*_ex_), the Dzyaloshinskii–Moriya strength (DMI, including bulk‐type DMI and interfacial‐type DMI), the saturation magnetization (*M*_sat_), and the anisotropy constants, respectively.^[^ ^3^ ^]^ If the key parameters are estimated properly, static and dynamical phenomena of artificial spin ice, Skyrmion, spin‐waves, and spintronics can be reproduced and predicted.^[^ ^4^, ^5^, ^6^ ^]^ Thus the Hamiltonian parameters estimation is essential in predicting and understanding properties of specific system.^[^ ^7^, ^8^, ^9^, ^10^, ^11^, ^12^, ^13^ ^]^

For magnetic systems, efforts have been devoted to extract their key parameters from the formation of a spin spiral using ferromagnetic resonance, Brillouin light scattering, or neutron scattering.^[^ ^14^, ^15^, ^16^ ^]^ However, these approaches hinge the inevitable measurements of time‐resolved dynamics and to do so locally. Consequently such detailed measurements and extensive post processing of the measured data render the estimation to be time‐ and cost‐consuming.^[^ ^14^, ^15^, ^16^, ^17^ ^]^ With recent advance in magnetic observing technique such as Lorentz transmission electron microscope, the experimental images are able to provide more detailed information of spin configurations. Although the spin configurations are determined by the magnetic Hamiltonian, extracting the exact values of these parameters from merely an image is not an easy task due to the huge Hilbert space.^[^ ^15^ ^]^ What is needed is a method that can automatically and appropriately estimate the Hamiltonian parameters from an experimental image.

Machine learning (ML) algorithms, such as tree‐based models,^[^ ^18^ ^]^ kernel‐based regressors,^[^ ^19^ ^]^ and artificial neural networks,^[^ ^20^, ^21^, ^22^, ^23^, ^24^, ^25^, ^26^, ^27^ ^]^ learn from the labeled data and predict the unexplored search space, providing a prevalent tool in condensed‐matter research. Examples of this include learning the phases and phase transitions of matters,^[^ ^20^, ^21^, ^22^, ^23^, ^28^ ^]^ solving the quantum many‐body problems,^[^ ^24^ ^]^ classifying the snapshots of ultracold atoms,^[^ ^25^ ^]^ estimating quantum parameter from quantum measurement,^[^ ^26^, ^27^ ^]^ decoding crystallography from high‐resolution electron imaging and diffraction datasets,^[^ ^29^ ^]^ classifying complex non‐collinear magnetic structures in 2D materials,^[^ ^30^ ^]^ and building low‐temperature phase diagrams.^[^ ^31^ ^]^ Given the success of machine learning in the classification problems in the examples shown above, we performed regression instead, which allows a quantitative estimation of the physical model parameters to reproduce the spin configuration and even to predict several material properties. Here we proposed an approach that trains ML model on images from numerical simulations and then applies the trained ML to achieve parameter estimation from an experimental image. A sliding window approach is introduced to decrease the number of training images, which significantly reduces the computational cost and renders an adaptive establishment of a ML model for a particular experiment image.

## 2 Strategy

**Figure 1:**
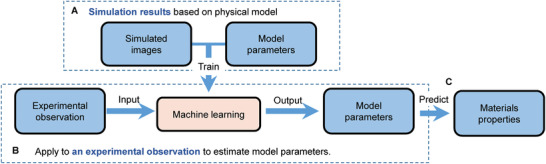
Flow chart of our approach. A) For a particular experimental observation, a training dataset is formed using simulated images of different Hamiltonian parameters under the same conditions as the experiment. A machine learning model is trained on these simulated images with labels of the Hamiltonian parameters, and consequently is capable to estimate Hamiltonian parameters from a new image. B) An experimental observation is input into the well trained machine learning model, which outputs the corresponding Hamiltonian parameters of that observation. C) The estimated Hamiltonian parameters can then be used to predict the properties of material.

**Figure** **1** shows the workflow chart of our approach. Given a particular experimental image, surrogate images are simulated under the experimental conditions by a physical or empirical model using different Hamiltonian parameters. A ML model is trained on these simulated images with labels of corresponding Hamiltonian parameters. Subsequently, the experimentally observed image is input into the trained ML model, which outputs the corresponding Hamiltonian parameters. The estimated Hamiltonian parameters are then used to predict the properties of material. Our approach allows us to estimate different Hamiltonian parameters simultaneously with a single input of experimental image, and without any other experimental measurement as prior knowledge. We demonstrate the efficiency of our approach by precisely estimating three key magnetic parameters (*A*_ex_, DMI, and *M*_sat_) from input of an experimental image. The real materials properties such as the magnetic hysteresis can be predicted and validated. Our work provides a new way to perform parameters estimation in an accelerated, accurate, and efficient manner.

It should be noted that our focus is on the soft magnetic material with non‐trivial spin texture, especially skyrmions with DM interactions. Thus we have ignored the magnetic anisotropy. However, our approach is applicable for estimating the full parameters as long as enough training data are collected, which consumes much simulation time to sample all parameters. For simplicity, the Hamiltonian parameters concerned here are *A*_ex_, DMI, and *M*_sat_. We combine micro‐magnetic simulation together with a convolutional neural network (CNN) to realize the estimation flowchart as shown in Figure 1.

The first stage is the preparation of the training dataset for CNN. The dataset presumably contains images of spin configurations and their corresponding magnetic parameters, as shown in Figure 1A. The value of *A*_ex_ is positive for ferromagnetism and negative for antiferromagnetism. There are two types of DMI including bulk‐type one for Bloch‐type skyrmions and interfacial‐type one for Neel‐type skyrmions. The dataset includes images covering values and different types of parameters to ensure a wild spread of labels. However, collecting such a dataset experimentally remains rather challenging as it is prohibitively laborious and expensive. Borrowed the idea from transfer learning of the robot training,^[^ ^21^ ^]^ we generate a training dataset containing simulated spin configurations utilizing micro‐magnetic simulation. Several discrete values of *A*_ex_ from ‐5 × 10^−11^ to 5 × 10^−11^ (J m^−1^), DMI from 1 × 10^−3^ to 5 × 10^−3^ (J m^−2^) for both bulk‐type and interfacial‐type and *M*_sat_ ranges from 1 × 10^5^ to 5 × 10^5^ (A m^−1^) are used (see, Supporting Information for details).

**Figure 2:**
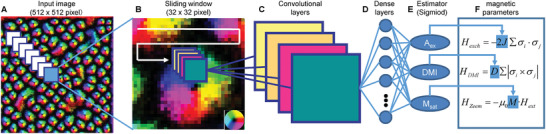
The implementation of a convolutional neural network. A) Input images of the spin configuration (each pixel can be considered as a spin, the color represents the spin orientation) which are labeled with magnetic parameters of *A*_ex_, DMI, and *M*_sat_. The overlapping sliding window on the input images enlarges the training observations. B) The slided small windows are input to a deep convolutional neural network with a variety of layers including C) convolutional filters, D) fully connected layers, and E) an output layer. The output layer is set as an estimator activated by sigmoid to output continuous values of parameters. F) The three neurons of final sigmoid layer output the values of magnetic parameters including *A*_ex_, DMI, and *M*_sat_. With those estimated parameters, one can predict materials behaviors and understand underlying physics.

In the second stage, a CNN architecture is established. The CNN architecture consists of convolutional layers and dense layers as shown in **Figure** **2**C,D, respectively. Unlike the traditional CNN that directly input the image to the convolutional layers, we introduce a data augmentation method of the overlapping sliding window to generate more input data on a small amount of simulated images before the convolutional layer, as shown in Figure 2A,B. An advantage of the physical system comparing to the inputs of conventional image processing is that the underlying information is distributed evenly, that is, any part of the image contains the same information from one set of model parameters. Thus we cut many portions of the input image by sliding the window, which serve as training images with their parameters known. This step greatly enlarges our training dataset and consequently leads to a better CNN (see, Supporting Information for details). Moreover, we replace the last layer of conventional CNN (usually a classifier) with an estimator by changing the active function from softmax to sigmoid. Doing so enables the CNN to estimate continuous values, as shown in Figure 2E. Specifically, the estimator layer includes three nodes, and each node will output a value of a particular magnetic parameter of *A*_ex_, DMI, and *M*_sat_ as shown in Figure 2F. By utilizing the trained CNN, these parameters for a particular spin configuration can be extracted and then the prediction of materials properties or the understanding of physics of magnetic phenomena by models such as micro‐magnetic simulation can be performed, as shown by Figure 1B,C.

## 3 Result

The efficiency of our approach and the performance of our CNN model are evaluated on both simulated test data and real experimental images.

### 3.1 Test on Simulated Images

**Figure 3:**
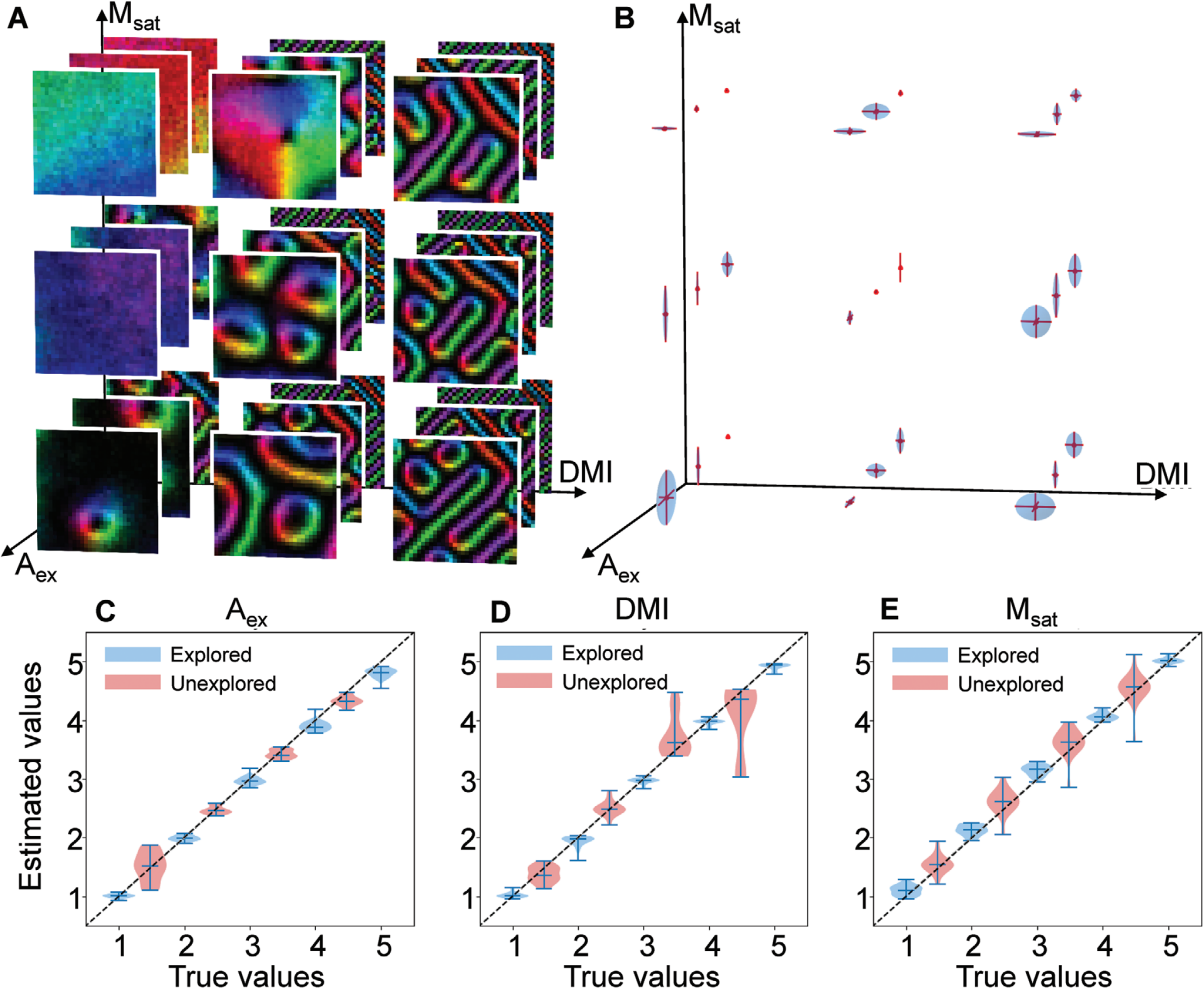
Test on simulation images. A) Spin configurations generated by micro‐magnetic simulation with the same parameter sets of *A*_ex_, DMI, *M*_sat_ used in the training data but with different initial random seeds. They possess different configurations compared with training data, but contain the same information of parameters. B) The estimation for the parameters from spin configurations in (A). Red points represent the true values of parameters used for simulating spin configurations in (A). Error bars in each direction indicate the deviation between estimation and the true values of the parameters. C–E) Plots of the estimated values as the function of true values of the *A*_ex_, DMI, and *M*_sat_, respectively. The blue ones are parameters used in the training data while the red ones represent parameters which are absent in the training data. The *y*‐axis values are statistics of estimations from 5 × 5 × 5 parameters sets. The upper and lower bars represent the quantiles 25% and 75%, and the central bar is the median.

For a clear illustration, we show only the results of simulated images belonging to chiral ferromagnetic system (*A*_ex_ > 0, and bulk‐type DMI) in the main text, and other situations are shown in Supporting Information. **Figure** **3**A shows a set of simulated spin configurations that are different from our training data but generated using the same set of magnetic parameters as the training spin configuration. The random initial seeds are different, thus different spin configurations were obtained. Inputting these unexplored configurations in Figure 3A to our trained CNN gives their magnetic parameters of *A*_ex_, DMI, and *M*_sat_. The estimated parameters are shown in Figure 3B, where the error bars in each direction indicate the deviation between estimation results and the true values of the parameters. It can be seen that there exists an error for these parameters estimations. For example, the estimation of *A*_ex_ is fairly good, and deviations of DMI and *M*_sat_ are slightly larger but still within an acceptable level. In Figure 3C–E, the estimated values of *A*_ex_, DMI, and *M*_sat_ were plotted as a function of true values in terms of the boxplot. The median, 25% quantile, and 75% quantile are shown by the short bars in the box‐plot. The estimated values and the true values follow a linear curve with the slope of 1, indicating a generalization estimation ability on all samples with different parameters sets.

We are more interested in the accuracy of the estimations when we apply the CNN to images with parameters never seen before. We generated 4 × 4 × 4 new spin configurations with parameters absent in the training dataset. As shown in Figure 3C–E by pink boxplots, the unexplored parameters are also around the diagonal line, revealing the robustness of our CNN. Therefore, our CNN model performs well for the testing dataset which is generated by micro‐magnetic simulation but has not appeared in the training process.

### 3.2 Test on Input Images with Different Sizes

**Figure 4:**
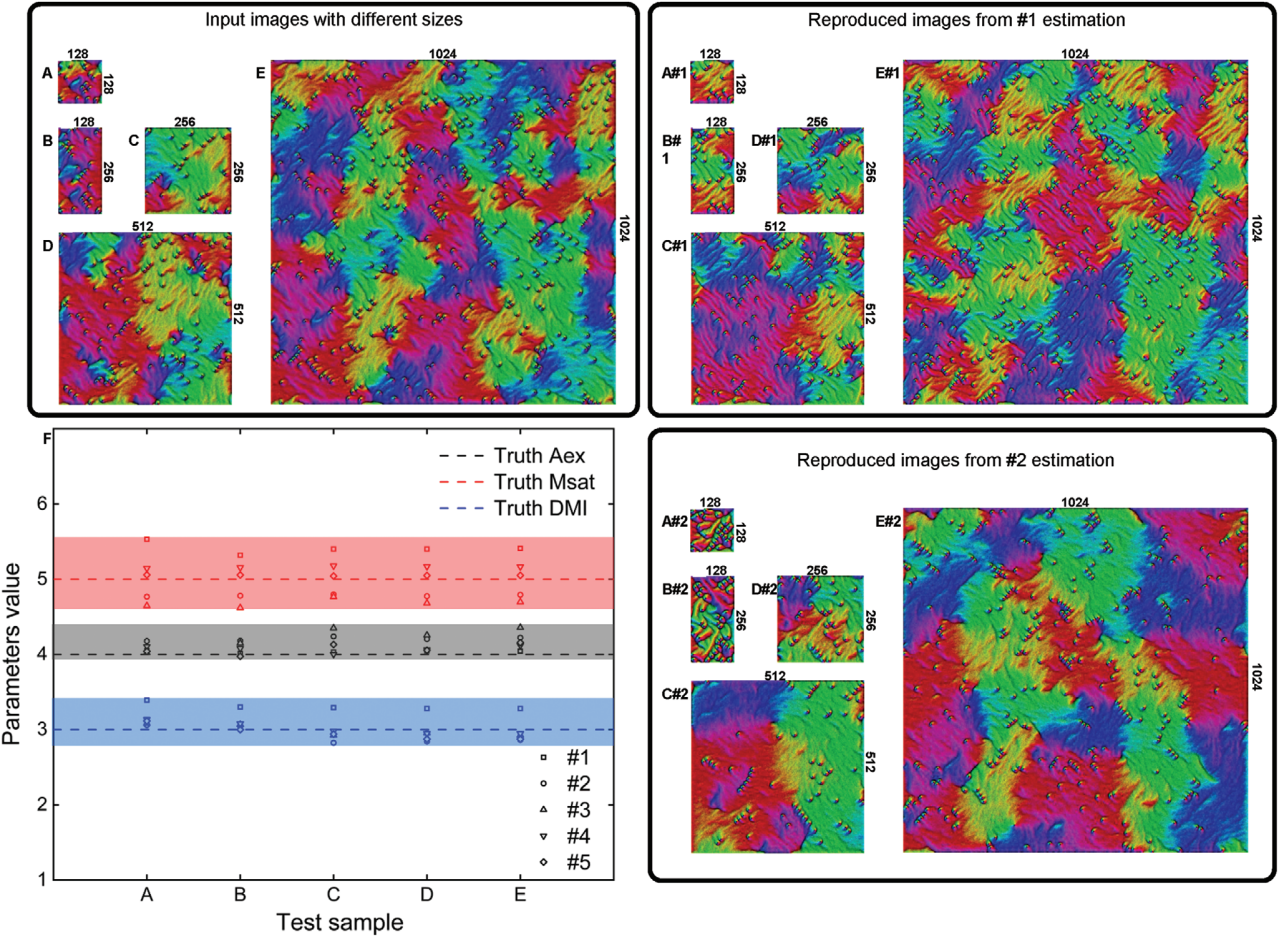
Test on different size images. A–E) Spin configurations from micro‐magnetic simulation with the same magnetic values of *A*_ex_ = 4(× 10^−11^ J m^−1^), DMI = 3(× 10^−3^ J m^−1^), and *M*_sat_ = 5 (× 10^5^ A m^−1^), but different image sizes of 128 × 128, 128 × 256, 256 × 256, 512 × 512, and 1024 × 1024. F) The results of five parallel trials (#1–#5). In each trial, we trained a new ML model and estimated a set of parameters. A#1–E#1) Spin configurations reproduced using the estimated parameters set #1. A#2–E#2) Spin configurations reproduced using the estimated parameters set #2.

The forecasting capability of our CNN is validated on above training and test datasets with same size of images. As the experimental observation varies in size, the generalization ability of our CNN to any size of image is of importance. To demonstrate such an ability, we estimate the parameters from input images with different sizes rather than only the size of 512 × 512 used in our training data. We generated five images of different sizes from the same set of parameters to be our test samples as shown in **Figure** **4**A–E. Noted that the morphology of spin configurations depends on the sample size,^[^ ^32^ ^]^ thus they are fresh to the CNN. We thus performed the estimation for five parallel trials. In each trial, we trained a new ML model and estimated the parameters using the same input image shown in Figure 4A–E. Therefore, five sets of parameters (#1–#5) can be estimated and these were plotted in Figure 4F, comparing with the true values. The CNN performs well regardless of the size of the input image, as the estimated values are distributed around the true values. However, there exist random errors due to the uncertainties during each ML training. For the estimated parameter set #1, the values of both *M*_sat_ and DMI are overestimated by 10%, while for the parameter set #2, the *A*_ex_ values are overestimated by ≈5% and the *M*_sat_ values are underestimated by ≈6%. But all the five sets of parameters can reproduce the similar spin configurations with the input ones. Figure 4 A#1–E#1, A#2–E#2 are the reproduced spin configurations from estimated parameter sets #1 and #2, respectively. To reduce the random errors in estimation, it is possible to use more training samples to train a bunch of parallel ML models and obtain the mean value of many trials.

### 3.3 Test on Experimental Images

**Figure 5:**
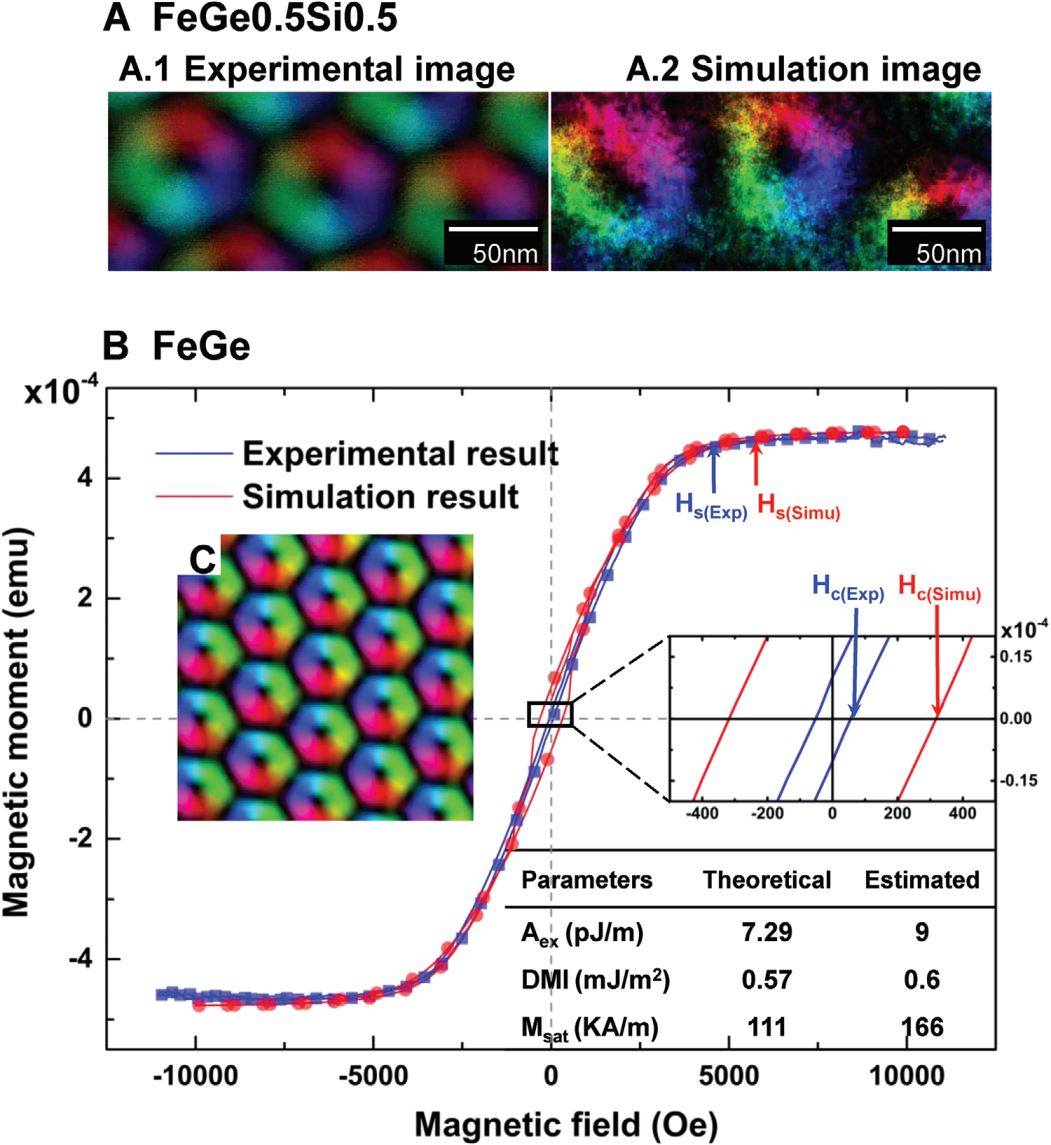
Test on experimental images. A) The comparison of spin configurations between the experimental image^[^ ^34^ ^]^ (the input to our CNN) and the simulated image using estimated parameters by our CNN. B) Inputting the spin configuration^[^ ^33^ ^]^ shown in (C) to our CNN, the parameters are estimated and comparable to the theoretical values^[^ ^36^ ^]^ as shown in the inset table. The hysteresis loop predicted by the micro‐magnetic simulation using these estimated parameters is in agreement with the experimental ones.^[^ ^39^ ^]^ The saturation field *H*_s_ is defined at the knee point in the *M*–*H* curve. The coercive field *H*_c_ is defined at the intersection point between loop and *x*‐axis.

With the power of estimating parameters from input images with different parameter sets, a key advantage of our CNN is its ability to be directly adapted to real experimental images. We chose FeGe^[^ ^33^ ^]^ and FeGe_0.5_Si_0.5_^[^ ^34^ ^]^ as test cases to validate our CNN model. These materials are of great interest due to the existence of the topological phase of skyrmions.^[^ ^35^ ^]^ We followed the workflow in Figure 1 to estimate three intrinsic parameters of *A*_ex_, DMI, and *M*_sat_. For each case of the two experimental observations, we generated a training dataset utilizing the corresponding experimental temperature and magnetic field to train a CNN. As we have the overlapping sliding window step, we did not need to generate a large amount of training data and consequently the process was rather efficient. The results of two examples are shown in **Figure** **5**. An experimental skyrmion lattice of FeGe_0.5_Si_0.5_ specimen by Lorentz TEM image reconstruction is shown in Figure 5A.1. The observation was performed at 95 K under 160 mT. Although the nominal composition of Si is 0.5, the actual composition is hard to determine and a precise estimation of parameters of this material is not an easy task. We generated a training dataset with 10 × 10 × 5 spin configurations by micro‐magnetic simulation at temperature of 95 K and under magnetic field of 160 mT. A CNN model was trained on this dataset. And then the magnetic parameters of *A*_ex_, DMI, and *M*_sat_ were estimated by inputting the experimental skyrmion lattice shown in Figure 5A.1. Using the estimated parameters, a spin configuration was reproduced from the micro‐magnetic simulation, as shown in Figure 5A.2. As can be seen from Figure 5A, the reproduced image has the same scale bar as that of the experimental image. The statistics on the two image shows that the average radius of the skyrmions (determined by selecting the brightest circle in the image, which indicates the in‐plane spins in the spin configurations) is 25.65 nm for the experimental results and 24.28 nm for the predicted results; the distance between the center of neighboring skyrmions is 82.76 nm for the experimental results and 97.73 nm for the predicted results. The reproduced configuration exhibits very similar features with the experimental one, indicating a good estimation of these magnetic parameters.

Besides reproducing the spin configuration, it is also possible to predict the macroscopic properties of a material from only an experimental observation. A skyrmion lattice of FeGe thin film is shown in Figure 5C. It was observed by Lorentz TEM at 265 K under 50 mT. Adaptively, we generated 10 × 10 × 5 spin configurations by micro‐magnetic simulation using temperature of 265 K and magnetic field of 50 mT and trained a new CNN to perform the estimation. As shown in the inset table of Figure 5B, the estimated parameters are in agreement with the theoretical values for this kind of material, which were obtained by microwave absorption spectroscopy.^[^ ^36^ ^]^ We further predicted the hysteresis loop of FeGe at the observation temperature of 265 K. Since the damping of magnetization is a rate at which it relaxes to equilibrium, the damping parameter could not be extracted from an experimental image. We used the theoretical value of damping parameter^[^ ^37^ ^]^ to model the hysteresis shown in Figure 5B. For more precise prediction of magnetic hysteresis, damping parameter^[^ ^38^ ^]^ should be calculated or measured. Moreover, the predicted *H*_c_ based on the ML model is ≈6 times of the experimentally measured *H*_c_.^[^ ^39^ ^]^ The reason is that *H*_c_ is not an intrinsic property, which is highly sensitive to defects, shape, and temperature. The saturation field (*H*_s_) of the predicted hysteresis loop, which is an intensive property of the material, agrees with the experimental value,^[^ ^39^ ^]^ as shown in Figure 5B. Since we are not able to get access to the volume of the experimental sample, we varied the sample volume in our simulation to fit the experimental value of the magnetic moment, which is an extensive property. So that we can estimate the actual volume of the experimental sample around 1 mm × 1 mm × 30 µm, which is reasonable for a SQUID measurement.

## 4 Discussion

Here, we proposed such an adaptive strategy that trains CNN on simulated images for a particular experiment image and then the trained CNN is applied to that experiment image to extract particular parameters. The accuracy of the estimation increases with the reduction of the number of parameters to be estimated. For example, *M*_sat_ can be accurately determined by bulk hysteresis measurements. Using known *M*_sat_ values will greatly improve the accuracy of the estimation. Besides, it consumes less computational cost to train the machine learning model. Whereas for 2D materials such as monolayer CrI_3_, the *M*_sat_ is hard to measure, but our approach enables us to estimate magnetic parameters including *M*_sat_. For those problems with *M*_sat_ known, our approach should perform even better.

Moreover, in the present study, it is from images under a non‐zero external magnetic field that the ML model can quantitatively extract the values of *A*_ex_, *M*_sat_, and DMI. If the external magnetic field is absent, only the ratio between *A*_ex_, *M*_sat_, and DMI determines the spin configuration, and thus only the ratio can be estimated. However, if a non‐zero external magnetic field is applied, the absolute values of *A*_ex_, *M*_sat_, and DMI matters. With the non‐zero external magnetic field known, absolute value of *M*_sat_ can be estimated from spin configurations and consequently the values of *A*_ex_ and DMI. It is also possible to include more tuning parameters which determine the spin configurations, such as the anisotropy and temperature. In these cases, a larger training dataset is required so that the trained CNN can be more general and applied to various experimental observations under different temperatures and with different shapes. However, it should be noted that from an experimental image it is impossible to extract parameters that do not determine the spin configurations, such as the damping parameter.

The mapping between the spin configurations and magnetic parameters are rather complex, for example, there are infinite possible configurations from one set of parameters due to the fluctuations and initial randomness. Traditionally, the manually designed descriptors to the spin configuration could inevitably loss part of useful information, which makes the estimation of parameters hard. The CNN which automatically designs as many descriptors of the spin configures as possible and extracts the most relevant features, is so far the best approach to handle this complex mapping problem. The above validations show that it is rather possible to acquire magnetic parameters from the spin configuration by a CNN machine learning algorithm.

The key ingredients of our approach include: 1) to overcome the shortage of well labeled experimental data, we train a CNN on a small training data with images generated by micro‐magnetic simulation, which is adaptable to a particular experimentally observed image under certain conditions such as sample shape, temperature, field, and resolution; 2) we propose a data augmentation method, that is, sliding initial image to effectively enlarge the number of input images, since the information of parameters distributed evenly throughout the whole spin configuration; and 3) we set the last layer of CNN to be an estimator for continuous values instead of the classifier for discrete ones.

## 5 Conclusion

In summary, we demonstrated a direct and efficient estimation of magnetic parameters from an experimental image via combination of numerical simulation and machine learning. Specifically, we demonstrated how to estimate targeted magnetic parameters via machine learning from only a single experimental image without any other experimental inputs. Such an adaptive feature allows us to deploy it to different experimental observations. The estimated parameters together with numerical simulations based on Hamiltonian can provide many information of the system, such as micrographies, macroscopic properties, phase diagrams, and so on. It is thus likely to accelerate the discovery of new materials such as skyrmions with the help of these predictions. Our approach provides a new paradigm to bridge theoretical Hamiltonian to the real material using the combination of numerical simulation and machine learning. It can be generalized to other condensed matter systems whose microstructure and properties are described by a Hamiltonian.

## 6 Experimental Section

##### Convolutional Neural Network

As the input image with parameters information distributed evenly, an overlapping sliding window method to enlarge the dataset was employed. A variety of data augmentation methods were studied and it was found that only the overlapping sliding window worked on the spin configuration. Other augmentation methods such as scaling and rotation would change the meaning of spin configurations. The overlapping sliding window method was found better than the non‐overlapping sliding windows, and the best window size equaled 32 pixels and the best slide step size equaled 8 pixels (see the Supporting Information for comparison between different window size and different step size). Such a setting could help CNN perform well while keeping it small and easy to train. Motivated by the success of CNN in image recognition, convolutional layers to extract parameters information by feature maps were employed. A variety of network architectures were studied and it was found that convolutional neural networks had much better performance than fully connected networks with the same number of layers. To achieve the estimation task, the last layer a sigmoid activation function was applied. The detailed architecture is defined in the Supporting Information.

During training, the parameters of the CNN were adjusted iteratively to minimize a cost function of mean‐square‐error (MSE). Stochastic gradient descent, along with back propagation, was used for lowering the cost function. The training was stopped and all parameters of CNN were set when the MSE saturated.

##### Test on Micro‐Magnetic Simulation

A GPU‐accelerated micro‐magnetic simulation program, MuMax^3^,^[^ ^40^ ^]^ was employed to generate spin configurations under different parameter sets and with different initial magnetization seeds. The magnetostatic energy was imbedded in the MuMax^3^, which was calculated by the saturation magnetization and lattice parameters. During estimation of the parameters by machine learning, the extracted parameter (*M*_sat_) together with the input of cell lattice size determined the magnetostatic energy.

To reduce the time cost of the simulation, a monolayer sample was simulated to approximate thin film sample or thin specimen used during the transmission electron microscope observation. But the idea was general and could be extended to more complex situations.

For the testing on simulated images, the environment parameters and geometry parameters were set as:

(1) Temp=300K

(2) $$\mathrm{Bext}=\left(0,0,0.18\right)$$

(3) $$\mathrm{setgridsize}\left(512,512,1\right)$$

(4) $$\mathrm{setcellsize}\left(4\times 10^{-9},4\times 10^{-9},1\times 10^{-9}\right)$$

The magnetic parameters were set as *A*_ex_ = −5, −4, −3, −2, −1, 1, 2, 3, 4, and 5 (× 10^−11^ J m^−1^), DMI = 1, 2, 3, 4, and 5 (× 10^−3^ J m^−2^) for both bulk‐type and interfacial‐type, and *M*_sat_ = 1, 2, 3, 4, and 5 (× 10^5^ A m^−1^). In total, there were 500 parameter sets. And 500 spin configurations under these parameter sets with an initial magnetization seed 1 were generated, as shown by blue color label train in Figure S2, Supporting Information.

In order to test, test_A dataset was first generated, which included 500 spin configurations under the above parameter sets but with a different initial magnetization seed of 2, as shown by green color label in Figure S2, Supporting Information. Furthermore, test_B dataset was generated, which contained 256 spin configurations under different parameter sets of *A*_ex_ = −4.5, −3.5, −2.5, −1.5, 1.5, 2.5, 3.5, and 4.5(× 10^−11^ J m^−1^), DMI = 1.5, 2.5, 3.5, and 4.5 (× 10^−3^ J m^−2^) for both bulk‐type and interfacial‐type, and *M*_sat_ = 1.5, 2.5, 3.5, and 4.5 (× 10^5^ A m^−1^), which were not explored by the CNN yet, as shown by yellow color label in Figure S2, Supporting Information.

##### Experimental Image

The experimental image of FeGe_0.5_Si_0.5_^[^ ^34^ ^]^ was kindly provided by Dr. Matsumoto. FeGe_0.5_Si_0.5_ was grown from a FeGe0.8Si0.2 ingot annealed at 900 °C for 100 h by conventional solid‐state reaction. A thin‐film specimen was fabricated from a bulk crystal using an ion slicer (EM‐09100IS; JEOL Ltd.). Before the observation, the thin film was further polished with a low‐voltage and low‐angle Ar ion beam milling apparatus (Precision Ion Polishing System II; Gatan Inc.) and an ion cleaner. For Lorentz TEM observations, a scanning transmission electron microscope (JEM‐2100F; JEOL Ltd.) equipped with a probe‐forming aberration corrector (CEOS GmbH) was used and a Schottky field emission gun was operated at 200 kV. The image used here was observed at 95 K under 160 mT, and the pixel size was 0.54 nm.

In the present study, micromagnetic simulation on a 1 nm film was used to generate images for training in order to save the computational cost, as it was reported that the spin configuration was fairly stable below a threshold in thickness. Simulations were performed on samples with the thickness varying from 1 to 40 nm to check the thickness dependence in our simulation. The results are shown in Figure S6, Supporting Information. Up to 40 nm, the spin configurations within each layer were similar as those of 1 nm. And the thickness of the TEM specimen used here was within this range of 40 nm. Thus the simulated images of 1 nm film could mimic the experimental TEM specimen. The work flow was demonstrated in such an approximate manner, however, for those situations requiring precise estimations, the simulation spin configurations with exact the same thickness as experimental specimen should be generated for training.

To estimate the parameter of FeGe_0.5_Si_0.5_ with such an image, MuMax^3^ was used to generate the training data with the environment parameters and geometry parameters setting as

(5) Temp=95

(6) $$\mathrm{Bext}=\left(0,0,0.16\right)$$

(7) $$\mathrm{setgridsize}\left(512,512,1\right)$$

(8) $$\mathrm{setcellsize}\left(0.54\times 10^{-9},0.54\times 10^{-9},1\times 10^{-9}\right)$$

The *A*_ex_ was positive and the DMI was bulk‐type since the Bloch‐type skyrmions appeared in the image. So the magnetic parameters were set as *A*_ex_ = 1, 2, 3, 4, 5, 6, 7, 8, 9, and 10(× 10^−11^ J m^−1^), DMI = 1, 2, 3, 4, and 5 (× 10^−3^ J m^−2^) for bulk‐type and *M*_sat_ = 1, 2, 3, 4, 5 (× 10^5^ A m^−1^). A CNN was trained on such a simulated dataset. Then it was deployed to the experimental image to estimate the magnetic parameters of FeGe_0.5_Si_0.5_. After getting the results of FeGe_0.5_Si_0.5_, the spin configurations were reproduced with the estimated parameters.

The experimental image of FeGe^[^ ^33^ ^]^ was kindly provided by Dr. Esser. A 1.5 µm film of FeGe was grown on Si(111) via MBE. Then, a cross‐sectional specimen with [1‐10] orientation was prepared using the standard FIB procedures, including 1) using FIB mill to remove material and leave a rectangular specimen normal to the surface of the substrate with nominal dimensions of 1–2 µm thick by 30–40 µm wide; 2) using the ion beam to cut around the sides and bottom of the specimen; 3) specimens were then transferred to a standard 3 mm TEM grid; 4) a high accelerating voltage of 30 kV was used for both milling and initial thinning at an incidence angle of ≈2° off of parallel, with final thinning finishing at an accelerating voltage of 5 kV to reduce potential ion‐induced damage until the thickness <50 nm; 5) the specimen was transferred to a low energy Ar ion mill to further reduce sample preparation‐induced damage. The image used here was observed at 265 K under 50 mT, and the pixel size was 2.34 nm. To estimate the parameter of FeGe with such an image, MuMax^3^ was used to generate the training data with the environment parameters and geometry parameters setting as

(9) Temp=265

(10) $$\mathrm{Bext}=\left(0,0,0.05\right)$$

(11) $$\mathrm{setgridsize}\left(512,512,1\right)$$

(12) $$\mathrm{setcellsize}\left(2.34\times 10^{-9},2.34\times 10^{-9},1\times 10^{-9}\right)$$

The *A*_ex_ was positive and the DMI was bulk‐type since the Bloch‐type skyrmions appeared in the image. So the magnetic parameters were set as *A*_ex_ = 1, 2, 3, 4, 5, 6, 7, 8, 9, and 10(× 10^−11^ J m^−1^), DMI = 1, 2, 3, 4, and 5 (× 10^−3^ J m^−2^) for bulk‐type and *M*_sat_ = 1, 2, 3, 4, and 5 (× 10^5^ A m^−1^). A CNN was trained on such a simulated dataset. Then it was deployed to the experimental image to estimate the magnetic parameters of FeGe. After getting the results of FeGe, they were used to predict an *M*–*H* curve. The estimated *M*_sat_ was in unit of (A m^−1^) while the experimental magnetization was in unit of emu. Thus the volume of the experimental sample calculated was ≈3 × 10^−11^ m^3^. The calculated volume equaled 1 mm × 1 mm × 30 µm.

## Conflict of Interest

The authors declare no conflict of interest.

## Author Contributions

D.W. and S.W. contributed equally to this work. D.W., S.W., and D.X. came up with the idea. D.W. and S.W. carried out the micro‐magnetic magnetic simulation. D.W. and A.Y. provided the architecture of machine learning model. F.T. and K.C. provided the details about Lorenz TEM. D.X. and S.Y. supervised the project. D.X., D.W., S.W., S.Y., A.Y., Q.Z., C.Z., and Y.Z. substantially contributed to the interpretation of the results and the writing of the manuscript.

## Supporting information

Supporting Information
