# Supplementary material for: Machine Learning Magnetic Parameters from Spin Configurations
Source: Adv Sci (Weinh). 2020 Jul 1;7(16):2000566. doi: 10.1002/advs.202000566 (PMC7435232; doi:10.1002/advs.202000566)
Supplement: Supplementary file 1 — Supporting Information [file ADVS-7-2000566-s001.pdf]

((Supporting Information can be included here using this template))

Copyright WILEY-VCH Verlag GmbH & Co. KGaA, 69469 Weinheim, Germany, 2020.

# Supporting Information

## Supplementary material of machine learning magnetic parameters from spin configurations

*Dingchen Wang[1#], Songrui Wei[2#], Anran Yuan[3], Fanghua Tian[1], Kaiyan Cao[1], Qizhong Zhao[1], Yin Zhang[1], Chao Zhou[1], Xiaoping Song[1], Dezhen Xue[1*], Sen Yang[†]*

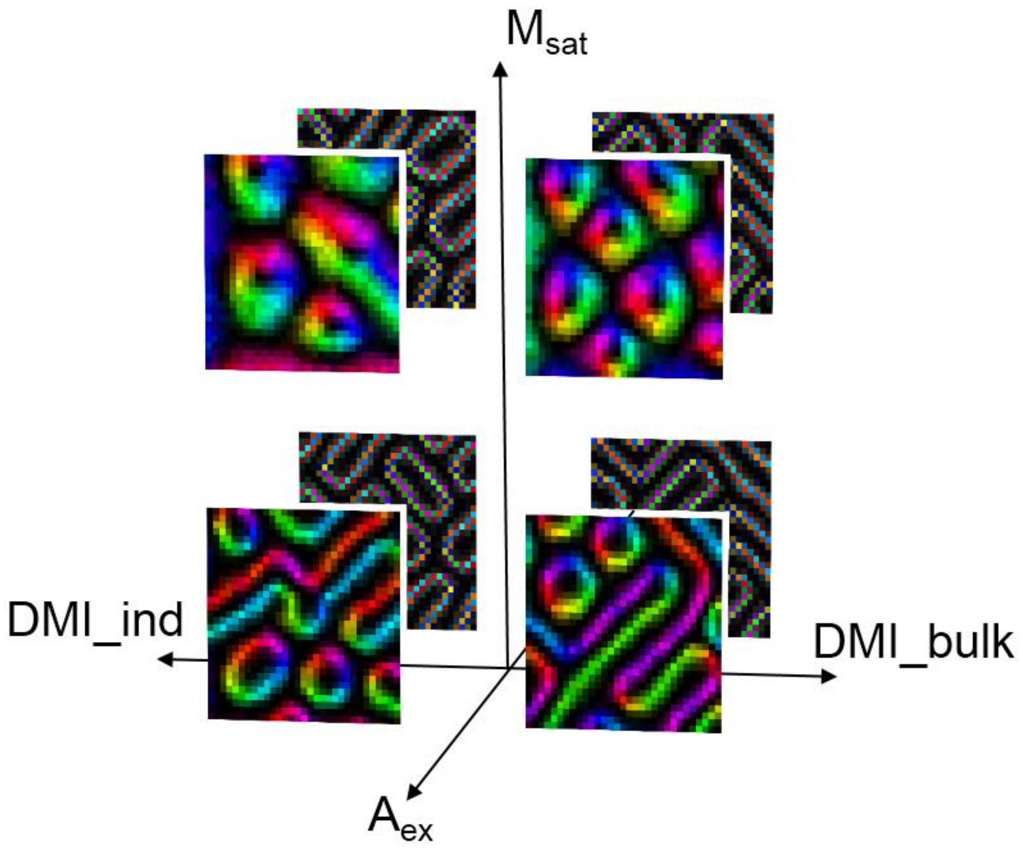


**Figure S1. Illustration of spin configurations in different system,** including all possible types of spin configuration whose $A_{ex}$ is positive or negative, *DMI* is bulk-type or interfacial-type.

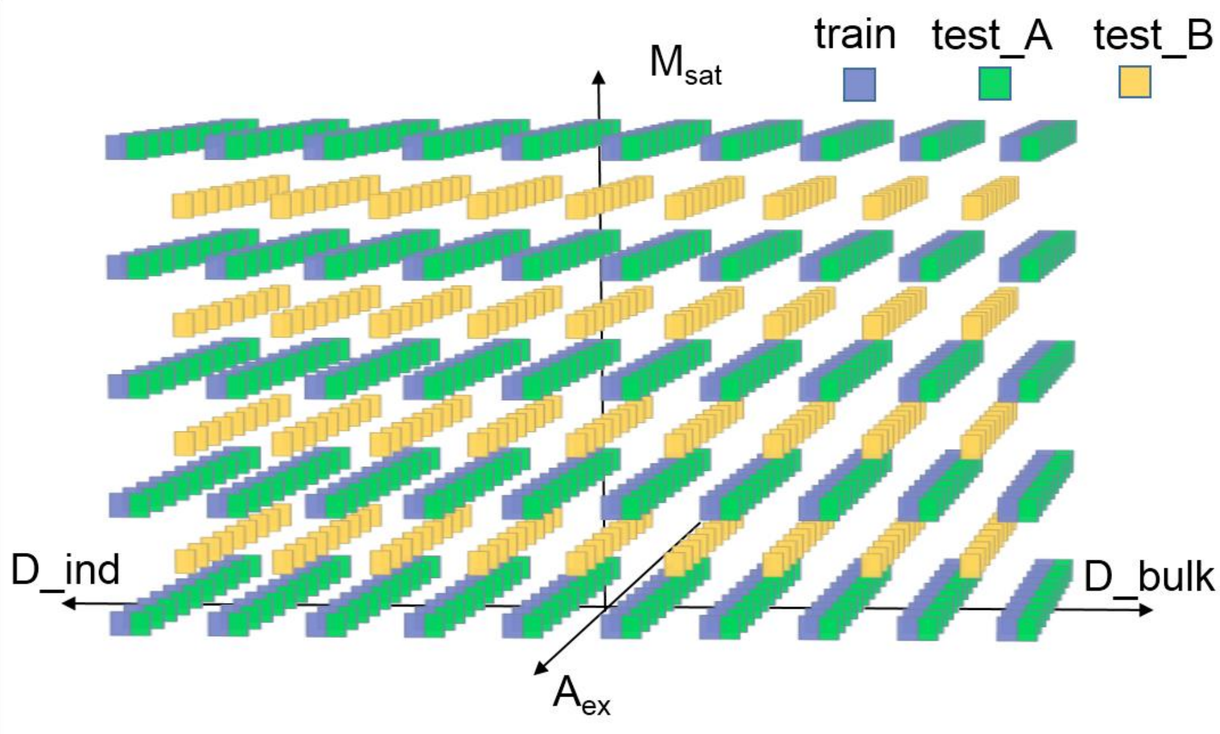


**Figure S2. The magnetic parameters used in the training set and test set.** Blue blocks represent training data, which were generated with an initial magnetization seed 1. Green blocks represent test_A data, which were generated with the same magnetic parameters as the training data but with an initial magnetization seed 2. Yellow blocks represent test_B data, which were generated from unexplored magnetic parameters.

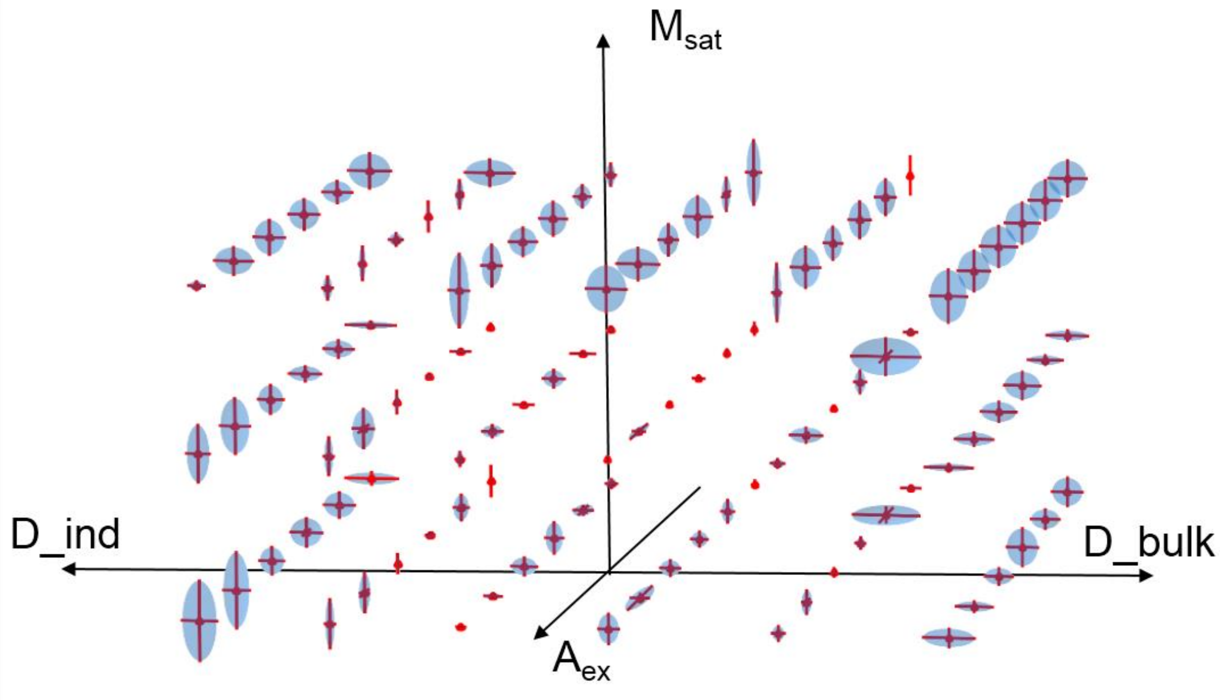

**Figure S3. Estimated parameters for spin configurations in different systems(test_A dataset).** Red points are the true values of magnetic parameters, error bars in each direction indicate the deviation between estimation and the true values of the parameters. The estimation is robust in all possible systems.

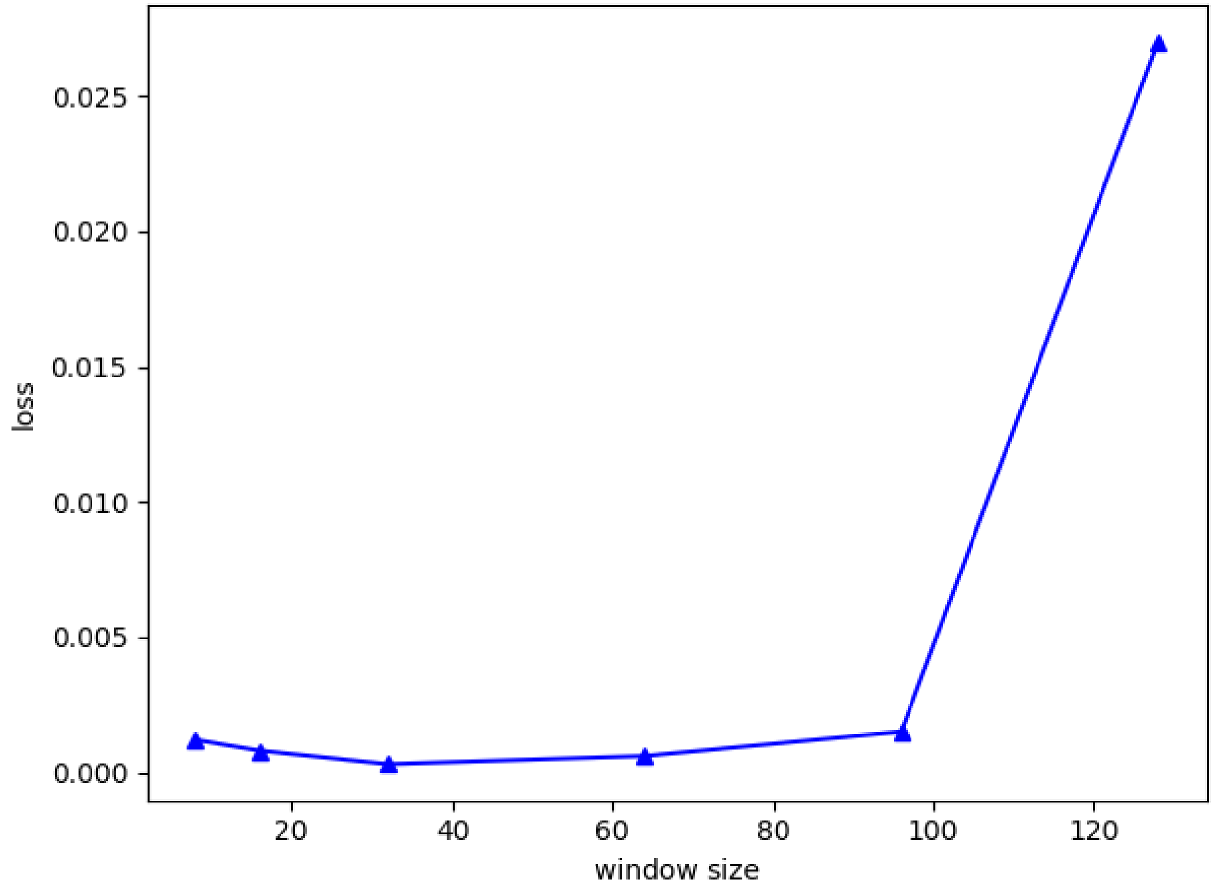


**Figure S4. Model loss at different window sizes.** The model loss function is minimized when window the size in overlapping sliding window layer of convolutional neural network is 32.

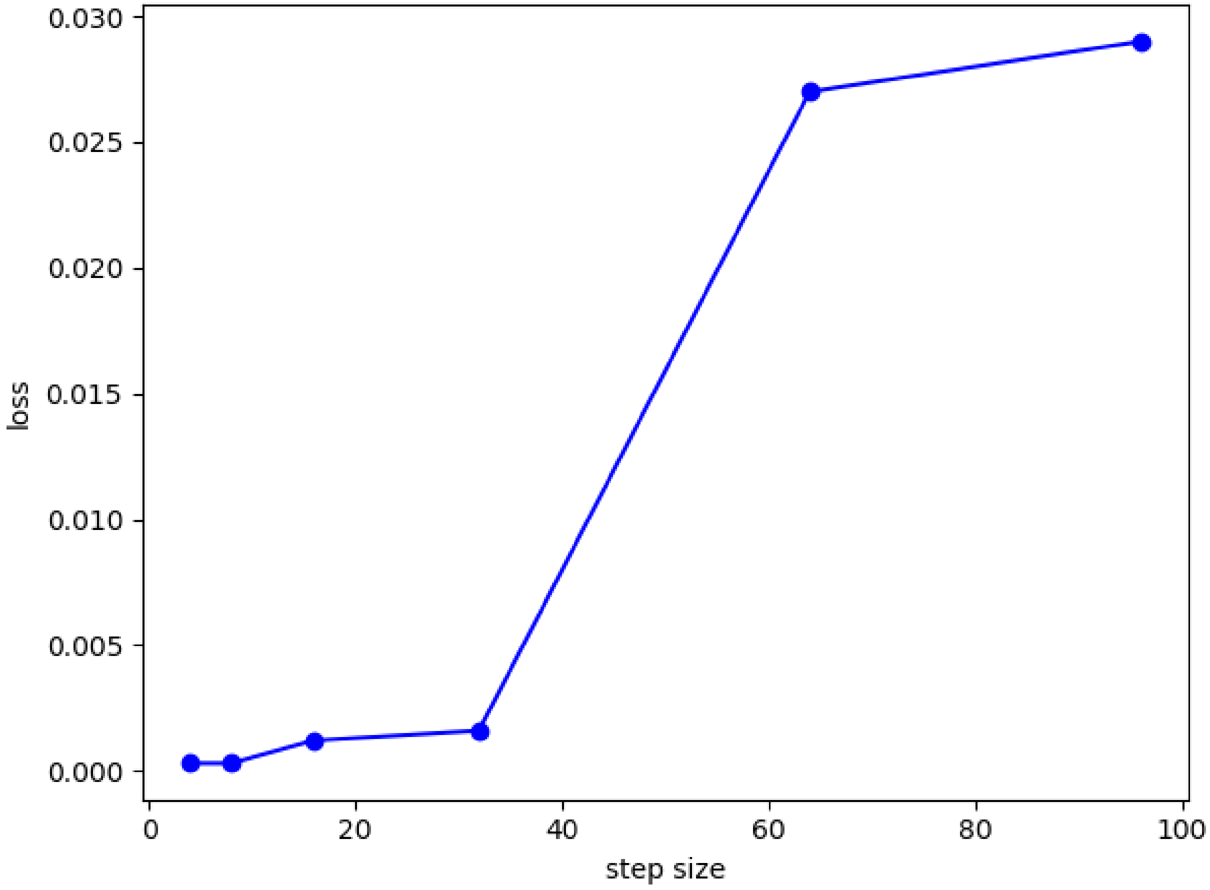


**Figure S5. Model loss at different step sizes.** The model loss function is minimized when the step size in overlapping sliding window layer of convolutional neural network is 4 or 8. The computation cost is less for step size 8, comparing with that for step size 4. The step size used is 8.

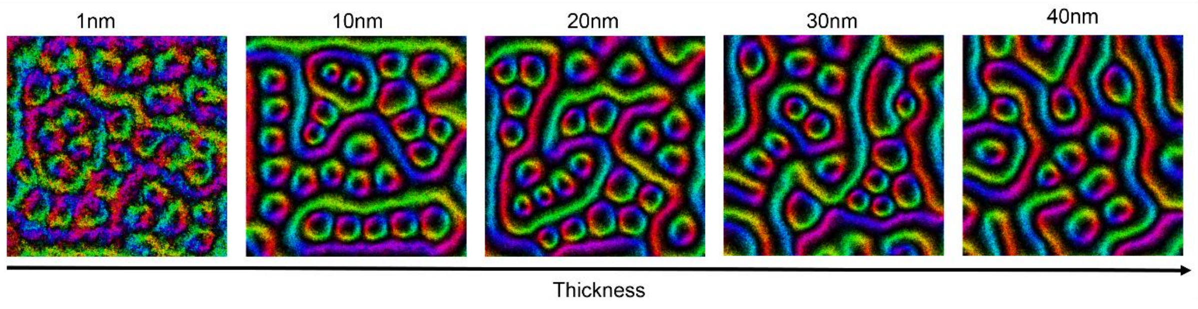


**Figure S6. Spin configurations at different thickness.** Up to 40nm thickness the resulting spin configurations within each layer are similar with those of 1nm thickness.

**Table S1. Convolutional Neural Network Architecture.**

| Layer | Layer name | Layer function | Layer description |
|---|---|---|---|
| 1 | original image | Image input | 512×512×3 image of PNG format |
| 2 | overlapping sliding window | Cut image | window size:32×32 sliding step size:8 |
| 3 | conv−1 | Convolution | 64 3×3×3 convolutions with strides |
| 4 | relu−1 | Relu | Rectified-linear unit layer |
| 5 | padding−1 | Padding | Zero padding |
| 6 | maxpooling | Maxpooling | Maxpooling |
| 7 | dropout−1 | Dropout | 25% dropout |
| 8 | conv−2 | Convolution | 128 3×3×64 convolutions with strides |
| 9 | relu−2 | Relu | Rectified-linear unit layer |
| 10 | padding−2 | Relu | Zero padding |
| 11 | dropout−2 | Dropout | 25% dropout |
| 12 | fc−1 | Fully connected | fc layer with 512 neurons |
| 13 | fc−2 | Fully connected | fc layer with 64 neurons |
| 14 | dropout−3 | Dropout | 50% dropout |
| 15 | sigmoid | Sigmoid | Sigmoid |
| 16 | estimator | Estimation output | MSE Loss |
